# Circulating and Brain BDNF Levels in Stroke Rats. Relevance to Clinical Studies

**DOI:** 10.1371/journal.pone.0029405

**Published:** 2011-12-16

**Authors:** Yannick Béjot, Claude Mossiat, Maurice Giroud, Anne Prigent-Tessier, Christine Marie

**Affiliations:** 1 U887 Motricité-Plasticité, Dijon, France; 2 Department of Neurology, University Hospital, Dijon, France; 3 Dijon Stroke Registry, EA 4184, Dijon, France; 4 Université de Bourgogne, Dijon, France; The University of Sydney, Australia

## Abstract

**Background:**

Whereas brain-derived neurotrophic factor (BDNF) levels are measured in the brain in animal models of stroke, neurotrophin levels in stroke patients are measured in plasma or serum samples. The present study was designed to investigate the meaning of circulating BDNF levels in stroke patients.

**Methods and Results:**

Unilateral ischemic stroke was induced in rats by the injection of various numbers of microspheres into the carotid circulation in order to mimic the different degrees of stroke severity observed in stroke patients. Blood was serially collected from the jugular vein before and after (4 h, 24 h and 8 d) embolization and the whole brains were collected at 4, 24 h and 8 d post-embolization. Rats were then selected from their degree of embolization, so that the distribution of stroke severity in the rats at the different time points was large but similar. Using ELISA tests, BDNF levels were measured in plasma, serum and brain of selected rats. Whereas plasma and serum BDNF levels were not changed by stroke, stroke induced an increase in brain BDNF levels at 4 h and 24 h post-embolization, which was not correlated with stroke severity. Individual plasma BDNF levels did not correlate with brain levels at any time point after stroke but a positive correlation (r = 0.67) was observed between individual plasma BDNF levels and stroke severity at 4 h post-embolization.

**Conclusion:**

Circulating BDNF levels do not mirror brain BDNF levels after stroke, and severe stroke is associated with high plasma BDNF in the very acute stage.

## Introduction

Brain-derived neurotrophic factor (BDNF), which is mainly synthesized by neurons, is present in large amounts in the adult brain where it plays a crucial role in plasticity and function [1]. Blood also contains BDNF, which is at a higher concentration in serum than in plasma since platelets release BDNF during the clotting process [2]. Indeed, platelets which cannot synthesize BDNF are rich in BDNF because they are able to take up the BDNF present in plasma. However, the cellular source of the BDNF present in plasma is not known. BDNF may be secreted into the plasma by endothelial or circulating immune cells [3]–[6]. From the evidence of parallel changes in serum and cortical brain BDNF during postnatal development in rats [7], the brain has been assumed to be an additional source of the BDNF present in plasma. Nevertheless, recent studies have shown that changes in regional brain BDNF levels are not associated with respective changes in plasma or serum [8]–[10].

Brain BDNF has been extensively studied in animal models of ischemic stroke. The studies consistently showed that brain BDNF levels increased after stroke [11]–[13], suggesting that BDNF promoted post-lesional plasticity [12], [14], [15]. A few studies have focused on BDNF in stroke patients [16]–[19]. In these studies, neurotrophin levels were measured in blood samples. In the interpretation of circulating BDNF levels it was assumed that BDNF levels increased in the brain of stroke patients as observed in animals and that circulating BDNF levels mirrored brain BDNF levels. However, BDNF levels have never been measured in post-mortem brains of stroke patients. Moreover, an unresolved but important question is whether circulating BDNF levels are modified by stroke. Indeed, circulating BDNF levels before hospital admission are unknown in patients, and there are no studies on circulating BDNF levels in animal models of stroke.

In order to help the interpretation of circulating BDNF levels in stroke patients, we measured BDNF levels in plasma, serum, and brain before and up to 8 days after the induction of embolic stroke in rats. This study, which is the first to document circulating BDNF levels in an animal model of stroke, was designed to answer the following questions: 1) does stroke induce changes in circulating BDNF levels? 2) do circulating BDNF levels mirror brain BDNF levels after stroke?

## Materials and Methods

All experimental procedures were performed on laboratory animals in accordance with institutional guidelines for the care and use of laboratory animals and were approved by an official committee. Adult male Wistar rats (290–350 g) were housed five per cage at 21°C in an artificial 12-h light and 12-h dark cycle with lights on at 7 am, and food and water *ad libitum.*

### 1) Induction of ischemic stroke

Multifocal infarction of the left hemisphere was induced in the rats by injection into the left carotid circulation of 50 µm-calibrated microspheres (carbonized microspheres, 3 M, Cergy-Pontoise, France), a model that is routinely used in our laboratory [13], [20]–[24]. In order to mimic the different degrees of stroke severity observed in patients, we chose this model that offers the unique opportunity to modulate stroke severity voluntarily by changing the number of injected microspheres, and that results in increased BDNF content of both hemispheres, but with a greater and more sustained increase in the lesioned than in the unlesioned hemisphere [13]_._ Animal death induced by embolization is in proportion with the number of injected microspheres and occurs mainly overnight after embolization. Briefly, after anaesthesia with chloral hydrate (400 mg/kg, i.p.) and definitive occlusion of the external carotid artery in order to prevent embolization of extracerebral tissue, a suspension of 2000, 3000 or 4000 microspheres in polyvinylpyrrolidone (20% in water) was injected (0.3 ml for 25–30 s) into the left common carotid artery. Then, the artery was definitively occluded. The embolized rats were assigned into one of the three time groups (“stroke 4 h”, “stroke 24 h” and “stroke 8 d”) according to the delay (4 h, 24h and 8 d) between embolization and brain removal. Each group included rats embolized with 2000, 3000 and 4000 microspheres.

### 2) Preparation of brain samples

The brains were collected after choral hydrate anaesthesia followed by transcardial perfusion with saline in order to flush blood out of the cerebral vasculature. The cerebellum, hypothalamus and mesencephale were discarded. The remaining hemispheres were homogenized in 7 volumes with ice cold buffer (pH 7.4) containing trizma base (100 mmol/L), NaCl (150 mmol/L), EGTA (1 mmol/L), triton (1%) and 1% cocktail of protease inhibitors. After centrifugation, tissue pellets were kept to assess the degree of embolization and supernatants were stored at −80°C until measurement of BDNF levels. When indicated, the remaining hemispheres were dissected in order to measure BDNF levels in the hippocampus, the thalamus, the striatum, and the entire cortex that were homogenized as indicated above.

### 3) Preparation of blood samples

Blood was withdrawn (by direct venous puncture) from the jugular vein, a vein that collects blood from the entire brain, before and after (4 h, 24 h, 8 d) embolization in the same rats. Two, 3 or 4 samples (1 ml each) were serially collected according to the time group of the stroke rats. The first and last samples were collected under chloral anaesthesia just before embolization and just before removal of the brain. Intermediate samples, if present, were collected under short-term halothane anaesthesia in order to reduce the overall duration of the anaesthesia. As the circadian rhythm may influence circulating BDNF levels [25], blood samples were always collected between 10 am and 2 pm. The blood was collected into sampling tubes and centrifuged to obtain serum or heparin-anticoagulated plasma. The plasma and serum were then divided into aliquots and stored at −80°C until BDNF measurements.

### 4) Assessment of the degree of embolization

As it is technically not feasible to simultaneously measure in the same rats the volume of infarcted tissue using histological method and the BDNF content of the whole brain, stroke severity was assessed from the degree of cerebral embolization. Indeed, we previously reported a strong correlation between the degree of cerebral embolization and clinically-pertinent markers of stroke severity including brain depletion in the neuronal marker N-acetyl-aspartate, vasogenic edema and acute neurological deficit [20]–[22]. In order to quantify the degree of cerebral embolization, the centrifugation pellets of brain tissue were dissolved in 5 ml of 20% KOH and centrifuged (500 g). The microspheres were then re-suspended in 0.5 ml KOH, and counted in triplicate in 50 µl of the suspension by a person blinded to the experimental conditions. A light microscope was used to count the microspheres.

### 5) BDNF measurement

BDNF levels were determined with a commercial sandwich ELISA kit (ChemiKine™, Cat. No CYT306, MERCK MILLIPORE). For this kit, rabbit polyclonal antibodies that were coated onto plates were generated against human BDNF and the captured BDNF were detected using biotin conjugated mouse monoclonal antibodies. BDNF antibodies do not cross with NGF, NT 4/5 or NT3. BDNF furnished by the kit to make the standard curve corresponds to the mature form of BDNF as revealed by an immunoblot analysis (data not shown). The limit of sensitivity was 6 pg/mL. The experimental procedure was performed according to the manufacturer's instructions. Supernatants of brain homogenates and serum samples were diluted (1/10, v/v) in the homogenization buffer (see above for its composition) and in TRIS buffer (pH 7.4). The diluted samples (50 µL) were again diluted (1/2, v/v) directly on the plate in a buffer provided by the kit. In contrast, plasma samples were not diluted (100 µL of plasma into each well). All assays were performed in duplicate. Brain BDNF levels were expressed as mg/g of fresh tissue and circulating BDNF levels as ng/mL

### 6) Statistical analysis

Data are expressed as mean ± standard deviation (SD). Statistical analysis was performed using non-parametric tests. The Kruskall Wallis test followed by the Mann–Whitney test was used to detect differences between groups of rats, and the Friedman's test followed by the Willcoxon's test to detect differences within a given group The Bonferroni's procedure was used to reduce the risk I error. The Spearman's rank correlation coefficient (r_s_) was calculated to examine the dependence between two variables. Significance was set at p<0.05.

## Results

Embolized rats (n = 81) were divided into the following groups: “stroke 4 h” (n = 15), “stroke 24 h” (n = 26), “stroke 8 d” (n = 20) and “stroke 8 d_s_” (n = 20). In the first three groups, blood samples were treated in order to obtain plasma while in the last group blood samples were treated in order to obtain serum. Serum and plasma BDNF levels were collected in separate groups of rats in order to avoid excessive blood withdrawal in a given rat. No mortality was observed in group “stroke 4 h”. In contrast, mortality reached 54% (14/26) in group “stroke 24 h” (2 rats died under halothane anaesthesia and 12 rats from embolization), 40% (8/20) in group “stroke 8 d” (1 rats died during halothane anaesthesia and 7 rats from embolization) and 40% (8/20) in group “stroke 8 d_s_” (2 rats died during halothane anaesthesia and 6 rats from embolization). In each group, we then selected surviving rats from the number of microspheres found in their brain so that the distribution of stroke severity was large but similar among groups. Accordingly, a certain number of rats were excluded from further experiments. Finally, 10 rats per group were selected, and the number of microspheres expressed as mean ± SD (minimal-maximal value) was 476±83 (328–615) in the “stroke 4 h” group, 412±122 (288–618) in the “stroke 24 h” group, 413±97 (271–554) in the “stroke 8 d” group, and 368±82 (242–487 for range) in the “stroke 8 d_s_” group. It was not surprising that the highest degree of cerebral embolization was lower in the groups “stroke 8 d”(554) and “stroke 8 d_s_” (487) than in groups “stroke 4 h” (615) and “stroke 24 h” (618) since highly embolized rats did not generally survive beyond 24 h. However, the distribution of stroke severity was large and quite similar in groups “stroke 4 h”, “stroke 24” and “stroke 8 d”. Brain BDNF levels measured in selected embolized rats were compared to those measured in control rats (no surgical procedure but anaesthesia and blood collection were on the model of group “stroke 8 d”, n = 7).

### 1) Regional distribution of BDNF in the brain

The regional distribution of BDNF was investigated in intact rats (no surgical procedure and no blood collection, n = 7). The results showed that the hippocampus contained the highest levels of BDNF (6.94±0.88 µg/mg) followed by the thalamus (5.32±1.19 µg/mg), the striatum (3.59±0.39 µg/mg) and the cortex (3.21±0.26 µg/mg). However, after consideration of the difference in fresh weight (mg) between the structures (68±5 for the hippocampus, 83±9 for the thalamus, 59±6 for the striatum and 402±22 for the cortex), it appeared that 50% of the total amount of BDNF found in the brain is cortical, the remaining BDNF being distributed in equivalent part between the hippocampus and other regions (thalamus and striatum).

### 2) Effect of stroke on circulating BDNF levels

**Figure 1:**
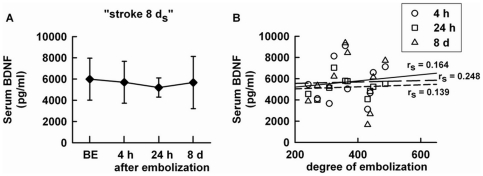
Serum BDNF after stroke. A) BDNF levels were serially measured before and 4 h, 24 h, 8 d after embolization in the “stroke 8 d_s_” group (n = 10), B) Scatter plot between individual BDNF levels and degree of cerebral embolization according to the time of measurement of BDNF levels after embolization. Data are expressed as mean ± SD, BE = before embolization, r_s_ = Spearman's rank correlation coefficient

**Figure 2:**
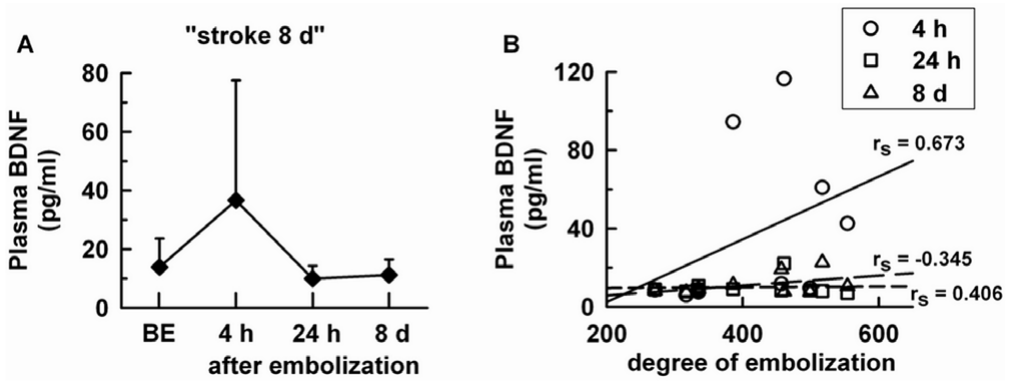
Plasma BDNF levels after stroke. A) BDNF levels were serially measured before and 4 h, 24 h, 8 d after embolization in the “stroke 8 d” group (n = 10), B) Scatter plot between individual BDNF levels and degree of cerebral embolization according to the time of measurement of BDNF levels after embolization. Data are expressed as mean ± SD, BE = before embolization, r_s_ = Spearman's rank correlation coefficient

The effect of stroke on circulating BDNF levels was assessed from the serial measurement of serum and plasma levels in the groups “stroke 8 d_s_” (Figure 1) and “stroke 8 d” (Figure 2), respectively. In these two groups, the distribution of stroke severity was quite similar (please compare individual degree of embolization between Figure 1 and Figure 2). Stroke did not modify serum BDNF levels (Figure 1A), and individual serum BDNF levels did not correlate with stroke severity at any time point after stroke (Figure 1B). Like serum levels, pre- and post-embolization plasma BDNF levels were not significantly different (Figure 2A). However, a positive correlation was observed between plasma BDNF levels at 4 h post-embolization and stroke severity (n = 10, rs = 0.673, p = 0.019) (Figure 2B). At other time points, no association was observed between the two parameters. The stroke severity-dependant changes in plasma BDNF levels at 4 h post-embolization coincided with the great inter-individual variability in plasma BDNF levels observed at this time point (please compare the standard deviation obtained at 4 h, 24 h and 8 d post-embolization in Figure 2A). Notably, a positive correlation between plasma BDNF levels at 4 h post-embolization and the degree of embolization was also found after pooling the groups “stroke 4 h”, stroke 24 h” and stroke 8 d” (n = 30, r_s_ = 0.364, p = 0.024). In these rats, pre- and 4 h post-embolization plasma BDNF levels were again not statistically different (22.2±16.5 and 37.8±44.6 ng/mL before and 4 h after embolization, n = 30, NS).

### 3) Relationship between brain and circulating BDNF after stroke

**Figure 3:**
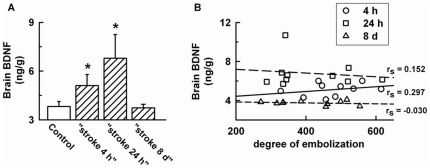
Brain BDNF levels after stroke. A) brain was collected in control rats (empty bars, 7 rats) and in the “stroke 4 h”, “stroke 24 h”, and “stroke 8 d” groups (dashed bars, 10 rats per group), B). Scatter plot between levels of BDNF in individual brains and degree of cerebral embolization. Data are expressed as mean ± SD, * significantly different from control rats (p<0.016), r_s_ = Spearman's rank correlation coefficient.

**Figure 4:**
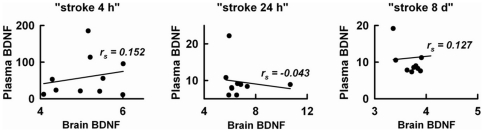
Scatter plot between individual levels of BDNF in plasma (ng/mL) and brains (mg/g) that were simultaneously measured 4 h, 24 h, and 8 d after embolization in the “stroke 4 h”, “stroke 24 h”, and “stroke 8 d” groups (10 rats per group), respectively, r_s_ = Spearman's rank correlation coefficient.

Figure 3A showed brain BDNF levels in intact and embolized rats (groups “stroke 4 h”, “stroke 24 h”, and “stroke 8 d”). Stroke significantly (Figure 3A) increased brain BDNF levels at 4 h and 24 h post-embolization, but the levels did not correlate with the degree of embolization in any of the three time groups of stroke rats (Figure 3B). When plasma BDNF levels were plotted against brain BDNF levels measured at the same time (4 h, 24 h or 8 d) after stroke we did not find any correlation between the two parameters (Figure 4).

As expected, no difference in brain BDNF levels (ng/g) was observed between groups “stroke 8 d” (3.74±0.22) and “stroke 8 d_s_” (3.36±0.68). When serum BDNF levels were plotted against brain BDNF levels, both parameters being measured at day 8 post-embolization, we did not find any correlation between the two parameters (n = 10, r_s_ = 0.139, NS, data not shown).

## Discussion

BDNF levels were measured in the whole brain and in the blood (plasma and serum) collected from the jugular vein in rats subjected to different severities of unilateral embolic stroke. Global rather than local brain BDNF levels were measured because the brain has been proposed to be a potential source of BDNF present in the blood. The measurements were made before stroke and up to 8 d after stroke. The results report an increase in brain BDNF levels at 4 and 24 h after stroke onset, which, however, was not accompanied by parallel changes in plasma or serum.

In the present study, embolic stroke was induced by the injection into the left carotid circulation of 50 µm-calibrated microspheres, a model that results in severe ischemia of the left hemisphere and formation of multiple infarcts that primarily affects the parieto-temporal cortex, the hippocampus and the thalamostriate areas [21], [22]. This model is unique because it allows inducing a large but controlled distribution of degrees of stroke severity by changing the number of injected microspheres. The present results showed that brain BDNF content increased after embolization and that the increase was not correlated with the degree of embolization. The lack of correlation between the two parameters supports our previous study revealing that the induction of BDNF synthesis in non-neuronal cells and BDNF over-expression by surviving neurons compensate for neuronal death-induced decreases in brain BDNF synthesis [13]_._

In stroke patients, circulating BDNF levels before hospital admission are unknown. Therefore, the effect of stroke on circulating BDNF can only be assessed by comparing BDNF levels in stroke patients with those in healthy subjects. However, difference between the two populations in life style, living environment and indices of metabolic and cardiovascular health, which are determinants of both plasma and serum BDNF levels [26], [27] may be confounding factors. Our present animal study in which BDNF levels were measured before and after embolization in the same rats offers the unique opportunity to directly investigate the effect of stroke on circulating BDNF levels. Our results revealed that stroke did not significantly change plasma BDNF levels when stroke severity was not taken into account. In contrast, a positive correlation was observed between plasma BDNF levels and stroke severity in the acute stage of stroke (4 h post-embolization). At the first sight, these data contrast with a study conducted on a small cohort (n = 10) of stroke patients [16]. This study reported a remarkable stability in plasma BDNF levels from hospital admission up to 4 d as well as a lack of correlation between plasma BDNF levels and the lesion size or the clinical score. However, it is possible that the first blood sample was collected too late in stroke patients (10.7 h±7.6 for SD vs 4 h post-embolization), thus missing the opportunity to detect stroke severity-dependent changes in plasma BDNF levels in patients. Alternatively, the lack of correlation may be due to a small number of patients with severe stroke (only two patients with a NIHSS >16, data on lesion size were not available). The mechanisms by which plasma BDNF levels at 4 h post-embolization are related to stroke severity have not been investigated here. However, the fact that plasma but not brain BDNF correlated with stroke severity supports the hypothesis that the excess of BDNF found in plasma of rats with severe stroke do not originate from the brain. An alternative but still speculative source of BDNF could be the endothelium. Indeed, circulating levels of pro-inflammatory cytokines or markers of oxidative stress at hospital admission have been previously correlated with stroke severity [28], [29]. Beside, when exposed to oxidative stress and pro-inflammatory cytokines endothelial cells synthesize and secrete high amounts of BDNF [3], [5]. Notably, cerebral endothelial cells express BDNF after brain embolization [13]_,_ and endothelium from the heart and the aorta shows strong basal BDNF immunoreactivity in rats (unpublished results of our laboratory)**.** Whereas plasma BDNF is taken up by platelets, the elevation of plasma BDNF levels at 4 h post-embolization did not lead to an increase in platelet BDNF content as evidenced by the lack of change of BDNF levels in serum after stroke. As BDNF levels are 200 times higher in serum than in plasma (in rats and humans), we suggest that severe stroke-induced elevation in plasma BDNF levels is too low to induce detectable changes in serum. Notably, it has been shown that serum BDNF levels were not associated with lesion size or recovery in stroke patients [17], [18]. Further clinical studies are needed to evaluate the reliability of plasma BDNF as an early biological marker of stroke severity.

The interpretation of circulating BDNF level in stroke patients has been made on the basis of the assumptions that circulating BDNF levels mirror brain BDNF levels. Our study that is the first to simultaneously measure circulating and brain BDNF levels in stroke rats reveal that changes in brain BDNF levels induced by stroke were not associated with parallel changes in plasma or serum. Thus, whereas stroke increased brain BDNF levels at 4 h and 24 h, it did not induce significant changes in plasma or serum. In addition, no correlation was observed between individual circulating and brain BDNF levels. These data clearly indicate that circulating BDNF levels do not mirror brain levels after stroke regardless the delay after stroke onset, thus challenging the idea that the measurement of circulating BDNF is a reliable way to assess BDNF levels in brain of stroke patients. The corollary is that that only post-mortem analysis of the ischemic brain will document the effect of human stroke on brain BDNF levels. Assuming that circulating BDNF levels do not reflect levels present in the brain after stroke, some clinical data may need to be reinterpreted. For instance, the stability in plasma BDNF levels observed in stroke patients [16] remain compatible with increased BDNF levels in the ischemic brain, and differences in serum BDNF levels that have been reported between patients without post-stroke depression (PSD) and PSD patients [18], [19] do not necessarily indicate that increased BDNF production in the brain of PSD patients is compromised.

A limitation of the present study is that the stroke model used lacks reperfusion after the initial blockage whereas reperfusion either spontaneous or induced occurs for a certain number of stroke patients. Therefore, our conclusion that circulating BDNF levels do not reflect brain BDNF levels in stroke patients could only concern patients lacking reperfusion. Nevertheless, even if reperfusion may be associated to the passage of BDNF from the brain into the blood, the detection in the blood of BDNF originating from the brain appears challenging because of a dilution effect of BDNF levels in the blood. Notably, a passage of BDNF from the brain to the blood is possible after embolization despite the lack of reperfusion as embolization is associated with both a disruption of the blood-brain barrier and a residual blood flow [20], [22].

In conclusion, stroke increases BDNF levels in the whole brain but not in the blood, and high plasma BDNF levels in the very acute stage are associated with severe stroke.
